# Enhancement of the Elastocaloric Performance of Natural Rubber by Forced Air Convection

**DOI:** 10.3390/polym16213078

**Published:** 2024-10-31

**Authors:** Emma Valdés, Enric Stern-Taulats, Nicolas Candau, Lluís Mañosa, Eduard Vives

**Affiliations:** 1 Departament de Física de la Matèria Condensada (FMC), Universitat de Barcelona, Martí i Franquès 1, 08028 Barcelona, Catalonia, Spain; evaldes@ub.edu (E.V.); enric.stern@ub.edu (E.S.-T.); lluismanosa@ub.edu (L.M.); 2 Institut de Nanociència i Nanotecnologia (IN2UB), Universitat de Barcelona, Diagonal 645, 08028 Barcelona, Catalonia, Spain; 3 Departament de Ciència i Enginyeria de Materials (CEM), Escola d’Enginyeria Barcelona-Est (EEBE), Universitat Politècnica de Catalunya BarcelonaTech (UPC), Av. Eduard Maristany 16, 08019 Barcelona, Catalonia, Spain; nicolas.candau@upc.edu; 4 Institute of Complex Systems (UBICS), Universitat de Barcelona, Martí i Franquès 1, 08028 Barcelona, Catalonia, Spain

**Keywords:** elastocaloric refrigeration, natural rubber, forced convection, Newton heat transfer coefficient

## Abstract

We study the enhancement of the elastocaloric effect in natural rubber by using forced air convection to favour heat extraction during the elongation stage of a stretching–unstretching cycle. Elastocaloric performance is quantified by means of the adiabatic undercooling that occurs after fast removal of the stress, measured by infrared thermography. To ensure accuracy, spatial averaging on thermal maps of the sample surface is performed since undercooled samples display heterogeneities caused by various factors. The influence of the stretching velocity and the air velocity is analysed. The findings indicate that there is an optimal air velocity that maximises adiabatic undercooling, with stretching velocities needing to be high enough to enhance cooling power. Our experiments allowed the characterisation of the dependence of the Newton heat transfer coefficient on the air convection velocity, which revealed an enhancement up to 600% for air velocities around 4 m/s.

## 1. Introduction

Many current efforts to develop environmentally friendly refrigeration are based on the elastocaloric effect in solids [1]. Very recently, there have been promising developments in prototypes using shape memory alloys and a regenerative technique, which feature very large cooling powers and outstanding temperature spans of over 50 K [2,3,4]. While these cooling performances largely overcome those reported for any other solid-state caloric technique [3], they require the application of large stresses, which are challenging to achieve. In contrast, natural rubber (NR) has been found to exhibit giant elastocaloric effects at a very low applied stress by virtue of its strain-induced crystallisation transition [5,6,7,8,9], which makes this material an excellent candidate for high-efficiency elastocaloric devices. When the NR polymeric chains are stretched, they show a collective behaviour that favours their arrangement into small regions of crystallites with significantly lower entropy compared to that in their disordered and unstretched state. In these conditions, releasing the stress adiabatically from the crystallised state results in large sample undercooling due to the reverse amorphisation transition [6,10].

Adiabatic temperature changes below −10 K have been reported when stress is released from a previously stretched sample under uniaxial forces at room temperature. Moreover, when samples are stressed by both uniaxial forces and twisting, maximum undercoolings ranging from −12 K to −68 K have been reported [11,12].

The development of NR-based elastocaloric refrigerators [13,14,15] faces various challenges. The main ones are due to the low thermal conductivity of NR, which constrains the high-frequency operation of a cooling cycle. In a simple cyclic refrigerator, the environment (air or water at room temperature) functions as a hot reservoir, and heat is extracted from the NR refrigerant to the environment during isothermal stretching or after adiabatic stretching. Ideally, the low-entropy crystallised state is maximised when elongated at room temperature. When the process is reversed, stress is released adiabatically and the sample cools down. Finally, the cold NR is put in contact with the cold reservoir to extract heat from it. This allows the refrigerant to revert to its original amorphous state at room temperature and start the cycle again. Recently, a comparison between samples with varying thicknesses evidenced that the slow heat transfer between rubber and air during natural convection delays the kinetics of strain-induced crystallisation [16], which might have a big impact on the elastocaloric performance of future refrigeration devices. Moreover, it has been suggested that adding carbon nanotubes or other fillers to elastomers can enhance the heat transfer from rubber to the environment [17]. To assess the impact of these enhancements on the elastocaloric performance, it is crucial to determine the heat transfer coefficient of pure NR.

Heat transfer from NR to air was studied 25 years ago using unstressed hot cylindrical samples [18,19]. In these works, assuming Newtonian behaviour, the authors considered a linear relation:

(1) $$\frac{dQ}{dt}=-hA\Delta T$$

where ΔT is the temperature difference between air and rubber, dQ/dt is the rate of heat loss, *A* is the sample surface and *h* is the heat transfer coefficient. The transient evolution of the temperature in the centre of the sample during cooling in air was compared to that in finite element simulations. The most accurate comparison yielded h≃ 17.4  W  $m^{-2}$  $K^{-1}$. To our knowledge, there is no available data on heat transport coefficients for samples that have been heated or cooled by internal elastocaloric effects and/or forced air convection.

In this work, we examine the heat extraction from NR during the stretching stage of the cycle when in contact with air at room temperature. We conduct a comparative analysis of natural and forced convection and also delve into the impact of stretching velocity. Our end goal is to enhance the elastocaloric effect, measured as the adiabatic undercooling that occurs following a fast stress release from the elongated crystallised phase.

## 2. Materials and Methods

### 2.1. Sample

The natural rubber of this study is SMR (Standard Malaysian Rubber) CV60 (Mooney Viscosity ML 1 + 4, 100 °C: 55–60), supplied by the company Akrochem (Akron, OH, USA), with 0.15% of hydroxylamine added to the latex stage to prevent the raw rubber from stiffening while storing. The NR was masticated inside the chamber of an internal mixer (Brabender Plastic-Corder W50EHT, Brabender GmbH & Co., Duisburg, Germany) at a temperature of 80 °C for 5 min and a rotation speed of 40 rpm. After 10 min, the vulcanising agent dicumyl peroxide (DCP) was added (1.5 wt% of the NR) and mixed for 5 min. The masterbatch containing NR and DCP was vulcanised according to the estimated optimal time at 170 °C [20] under 4 MPa. In order to perform the elastocaloric test, flat dogbone-shaped specimens were extracted from hot-moulded sheets by die-cutting with a specimen preparation punching machine (CEAST). The dimensions of the parallelepipedic sample neck, where crystallisation concentrates, along with the properties of the NR, are found in Table 1.

### 2.2. Experimental Setup

**Figure 1:**
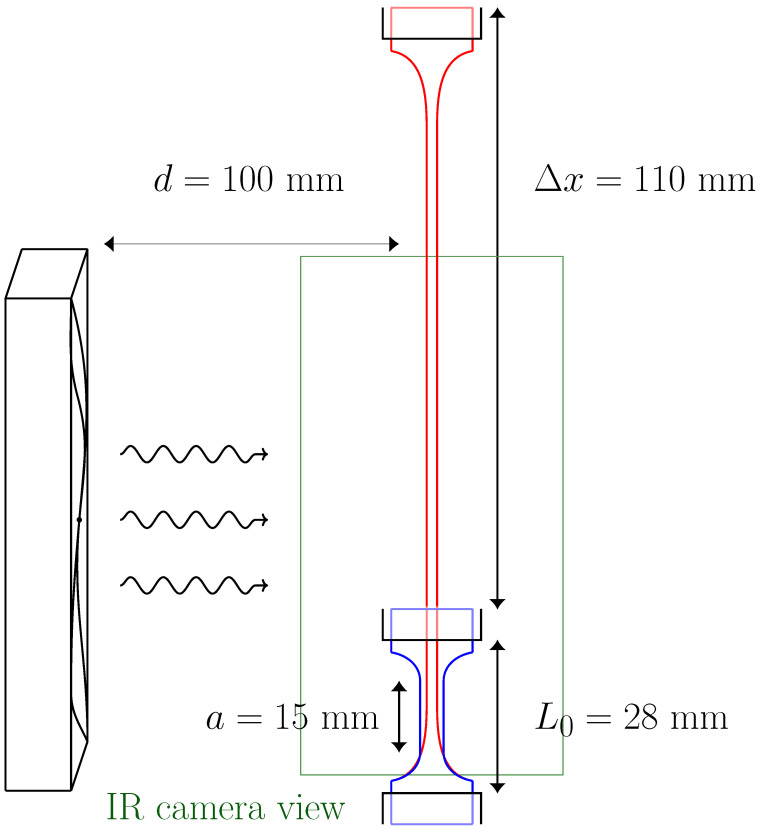
Schematic representation of the sample in both stretched and unstretched positions, as well as the location and size of the fan. The green rectangle indicates the area captured by the IR camera.

Stretching–unstretching experiments were carried out using a Zwick/Roell Z005 materials testing machine equipped with a 500 N load cell and an optical extensometer. Stretching protocols were performed with displacement control, and data were recorded at 10 samples/s. Figure 1 shows a schematic representation of the setup.

To capture thermal maps of the central and bottom parts of the sample surface, an Infratec 8800 infrared camera framing at a rate of 25 Hz was used. For the 250 × 512 pixel maps, the framing frequency was restricted to 5Hz. The force and displacement data from the materials testing machine was transferred to the IR camera software (IRBIS 3 Professional) for synchronisation. The camera lens was placed at 650 mm from the sample surface. The spatial resolution of the maps was 5.12 pixels/mm. The entire experiment was surrounded by a polystyrene cage to prevent inhomogeneities due to external infrared radiation reflections [21]. Ambient temperature was approximately constant at $T_{0}=22\pm 1\;{}^{\circ}C$.

For forced convection experiments, a 24 V brushless DC fan (Orion, OD9238-24HBVXC) was placed at a distance of 100 mm from the sample axis. Forced airflow was parallel to the 4 mm × 15 mm flat faces of the sample. The fan diameter was 90 mm, and its centre was at 45 mm from the sample base.

The fan was calibrated in a separate blank experiment by changing the applied voltage in the range 5 V–20 V and measuring the air speed $v_{\mathrm{air}}$ at the position of the centre of the sample using an anemometer. For this range of voltages, airspeed was in the range 0–5 m/s.

The sample was attached to the testing machine using steel grips. Prior to any experiment, the sample was always subjected to 5 conditioning cycles that imposed a grip-to-grip elongation of Δx=110 mm. After the conditioning protocol, the initial position of the grips for subsequent experiments was modified to have a zero-force reading (|F|≲0.01 N), resulting in an initial grip-to-grip length of $L_{0}≃28\;mm$.

### 2.3. Elastocaloric Cycling Protocol

**Figure 2:**
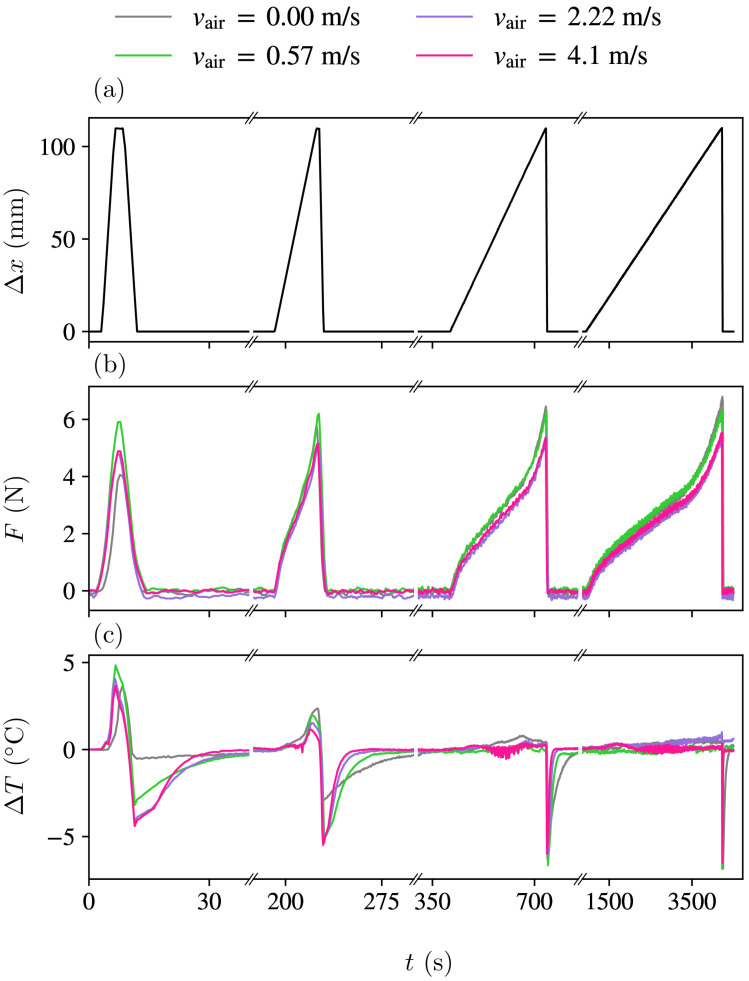
(**a**) Mechanical protocol consisting of four stretching steps (from left to right) at v= 2000, 200, 20 and 2 mm/min followed by fast unstretching at 2000 mm/min, with a 2 s pause in between. Long pauses of 180 s separate the cycles. (**b**) Corresponding behaviour of the applied force for each of the four air velocities $v_{air}$, as indicated by the line colour. (**c**) The behaviour of the temperature in the centre of the sample at each $v_{air}$. Note that the horizontal scale of these graphs is discontinuous and that each segment is plotted on a different time scale.

Figure 2a illustrates the stretching protocols, wherein a lengthening of Δx=110 mm is imposed at varying velocities. The experiment involved four rounds of stretching–unstretching cycles, each one with a brief 2-second pause. A long thermalisation pause of 1800 s was implemented between each cycle. The elongation ramps were carried out at stretching velocities of v= 2000, 200, 20 and 2 mm/min, and the unstretching ramps were always at high speed (2000 mm/min). During forced convection measurements, the fan was operated solely during the stretching process and was turned off at the beginning of the brief two-second pause prior to rapid unstretching. The goal was to minimise heat exchange during the unstretching process, resulting in maximum undercooling from enhanced adiabatic conditions.

The stretching ratio in the centre of the dogbone samples was λ=5.2 for elongations of Δx=110 mm, as determined independently in a separate experiment by tracking paint marks (separated by 13 mm) with the optical extensometer. It should be noted that this value differs from the commonly used total stretching ratio $\lambda^{\mathrm{total}}=\frac{a+\Delta x}{a}=8.33$, which is computed assuming that only the neck of the dogbone sample is elongating. The difference in these two values evidences that the heads of the sample contribute significantly to the total elongation. In this work, we use λ=5.2.

In Figure 2b, the measured force is plotted against time for various scenarios at selected loading velocities *v* and air velocities $v_{air}$. It is evident that the force required to reach the elongated state varies as a function of both parameters *v* and $v_{air}$.

### 2.4. Sample Temperature Determination

**Figure 3:**
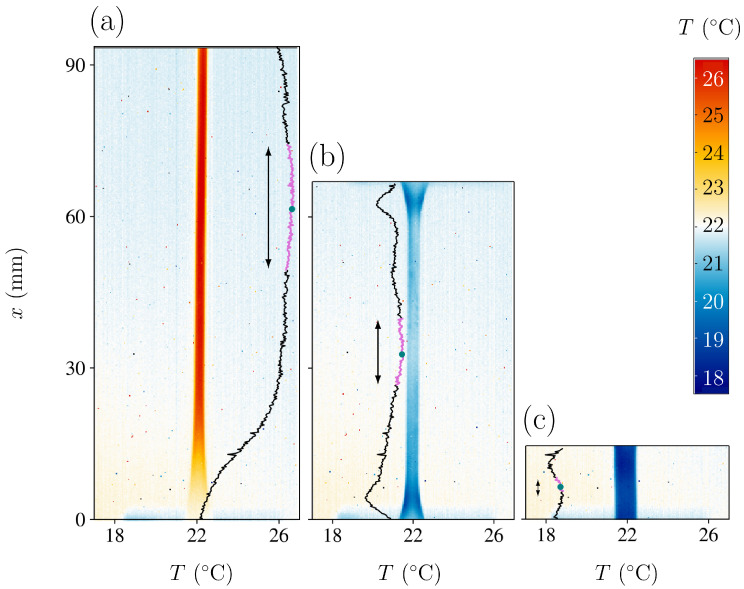
(**a**) IR camera view and central temperature profile of the sample at its maximum elongation. (**b**) IR camera view and central temperature profile in an intermediate position during the unstretching stage. (**c**) IR camera view and central temperature profile of the sample after returning to its initial position. On each temperature profile, we indicate in the colour violet and by using arrows the region used to determine the sample temperature T(t). The vertical length of this region corresponds to 20% of the total length L(t) of the sample.

Figure 3 shows three examples of temperature maps of the sample at $v_{air}=0.57\;m/s$ and v=2000 mm/min. Figure 3a displays the sample immediately after the stretching stage. The sample temperature is above room temperature due to the latent heat release during crystallisation. Figure 3b corresponds to an intermediate frame during the fast unstretching stage, and Figure 3c corresponds to the final frame after total stress removal. In the latter case, the sample is several degrees below room temperature due to the heat absorption in the amorphisation transition. Notice that in Figure 3a, the sample cannot be imaged at full length due to the limited field of view of the camera. However, the central region is always within the frame.

The black vertical curves indicate the temperature along the central vertical axis of the sample. The values can be read on the horizontal bottom axis. As can be seen, the sample temperature is not uniform, with regions displaying undercooling variations of more than 2 °C and temperature gradients larger than 0.5 °C/mm.

These temperature heterogeneities observed immediately after the unstretching stage may have resulted from stress heterogeneities within the stretched sample that locally affected the crystallisation degree. These, in turn, might be caused by differences in sample width, heterogeneities in sample composition, heat conduction towards the grips, heterogeneities in the air convection, etc. Even if convection is natural (without a fan), the displacement of the sample points in the upper part of the sample is faster than the displacement of the sample points in the lower part. Furthermore, when the experiment is performed with forced convection (the fan switched on), the airflow can also be inhomogeneous, depending on the fan speed.

For these reasons, to ensure an accurate measurement of the elastocaloric effect, we characterised the sample temperature *T* by averaging the central 20% of the neck length. For every snapshot in Figure 3, we indicated the central point (green dot) and the averaged segment in magenta. This protocol might yield lower undercooling values than other estimations reported in the literature, which may have characterised the elastocaloric effect by measuring undercooling at the point with the lowest temperature. In this regard, it can be noted that close to the ends of the sample, some regions display larger undercooling than the averaged central region.

A second possible source of errors in determining the sample temperature could be the change in the roughness of the NR surface when comparing the stretched and unstretched samples. The influence of roughness in emissivity is a well-studied effect in the literature [22,23]. To estimate the temperature variations of this effect, we measured the sample temperature in the two states (stretched and unstretched) after long waiting times at room temperatures. The temperature readings were consistent within a margin of ±0.3 K, which is comparable to the estimated room temperature fluctuations.

Figure 2c shows the difference between the average sample and room temperatures corresponding to each study case. Note that for low values of *v* and high values of $v_{air}$, the sample temperature remains nearly constant throughout the whole loading step, thus approaching an isothermal stretching protocol. On the contrary, for high stretching velocity *v* and $v_{air}=0$ (natural convection), the process is close to adiabaticity, and the sample shows high overheating during the loading step. In all cases, the high velocity of the unstretching step in the absence of forced air convection yields large undercooling.

## 3. Results and Discussion

**Figure 4:**
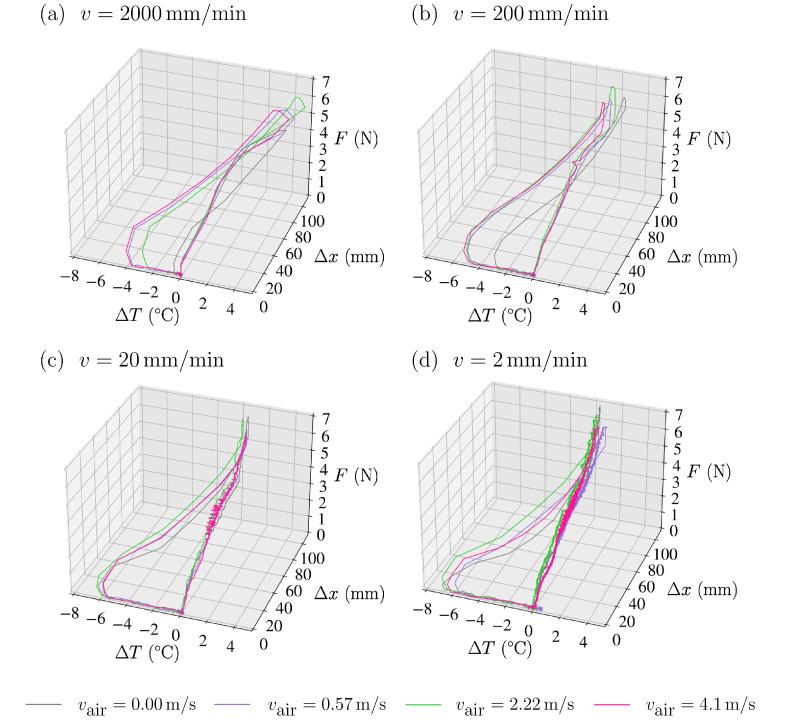
Applied force *F* (in N) vs. elongation Δx (in mm) vs. change in the average surface temperature ΔT of NR (in °C) for all loading–unloading cycles. The stretching velocities are (**a**) 2000 mm/min, (**b**) 200 mm/min, (**c**) 20 mm/min and (**d**) 2 mm/min. In all cases, unloading is at 2000 mm/min. The colour of each curve indicates the convective air velocity $v_{air}$, as shown in the legend below.

Given the out-of-equilibrium behaviour of rubber, the elastocaloric effect is better described by a three-dimensional trajectory in the F−Δx−ΔT diagram. The various analysed scenarios are depicted in Figure 4. Each plot corresponds to a different stretching velocity: (a) v=2000 mm/min, (b) v=200 mm/min, (c) v=20 mm/min and (d) v=2 mm/min.

The cycles start at $\Delta T=T-T_{0}=0$ and show an increasing *F* vs. Δx trajectory. Trajectories display a clear shift to high temperatures when stretched rapidly (a and b), which diminishes as $v_{air}$ increases. The overheating occurs approximately in the second half of the trajectory, which signals the region of large deformations where crystallisation occurs. In contrast, the trajectory remains rather isothermal regardless of the air velocity for the slow stretching cases (c and d).

Regarding the undercooling steps, which occur at a fast rate in the absence of forced convection, a significant undercooling is noticed by the trajectories moving towards the left side of the graphs. Higher levels of undercooling, reaching almost $\Delta T_{unstr}=-8\;{}^{\circ}C$, are attained when the sample is subjected to slow stretching and prolonged exposure to large deformations, which favours crystallisation.

It is worth noting that increasing $v_{air}$ values caused deeper undercooling for the fastest stretching experiments in which the sample remained deformed for shorter periods (panel (a) in Figure 4). In this regard, magenta and violet lines show undercooling of $\Delta T_{unstr}∼$ −6 °C, clearly more negative than the green and black lines. This is indicative that increasing heat extraction during fast stretching favours crystallisation.

### 3.1. Elastocaloric Adiabatic Undercooling

**Figure 5:**
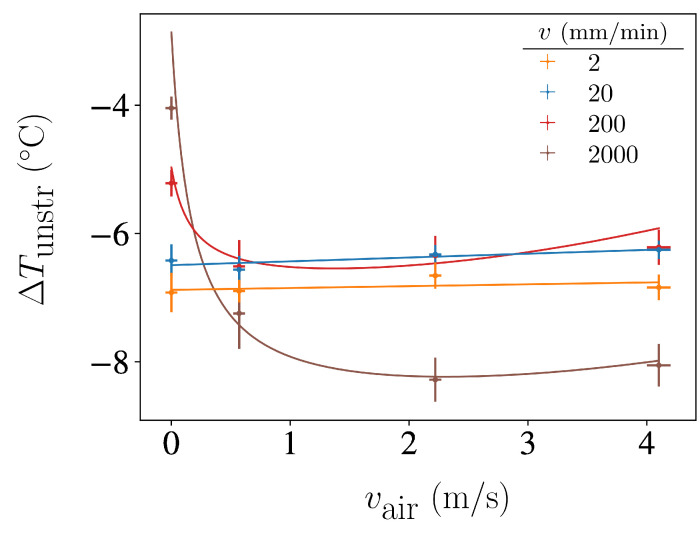
Maximum undercooling at the centre of the rubber sample achieved from rapid unstretching after stretching at different velocities *v*, as a function of the velocity of air convection $v_{air}$ during the stretching stage.

To better understand the effect of forced convection during the stretching stage, in Figure 5 we compile the values of the maximum undercooling values $\Delta T_{unstr}$ as a function of the air velocity $v_{air}$ for each stretching velocity *v*. As can be observed, forced convection enhances undercooling in the case of fast stretching experiments, while it has a nearly negligible influence in the case of slow stretching experiments. This suggests that there is competition between heat release and crystallisation growth when rubber is stretched fast. In the presence of this competition, forced convection can promote crystallisation. However, heat extraction no longer limits crystallisation if the stretching process is sufficiently slow.

The reason for the minimum in the curves for high stretching velocities is unclear. One possible explanation for the decrease in undercooling at the highest $v_{air}$ values is that turbulent airflow may negatively impact forced convection heat extraction efficiency.

The observed $\Delta T_{unstr}$ undercooling values range from −6 °C to −8 °C, which are slightly smaller (in absolute value) compared to those found in the literature for similar stretching ratios in natural rubber (see Table 2). This might be due to the fact that we report an average surface temperature rather than the temperature of the coolest region of the sample.

### 3.2. Newton Heat Transfer Coefficient

To quantitatively model the effect of air convection on the dissipation of internally generated heat at the crystallisation transition, we conducted experiments maintaining constant air velocity for a prolonged period after stretching or unstretching. Temperature evolution during these periods was analysed to derive the Newton heat transfer coefficient in each case.

Assuming that losses to air are the primary cause of thermal changes during such pauses at constant elongation, one can integrate Equation (1) to obtain an exponential time evolution for the temperature:

(2) $$T\left(t\right)=T_{0}-\left(T_{0}-T_{i}\right)\cdot e^{-t/\tau },$$

where $T_{0}$ is the ambient temperature, $T_{i}$ is the initial temperature immediately following either stretching or unstretching, and *t* is the time elapsed since the pause has begun. The time constant τ that can be fitted from the experimental data is given by

(3) $$\tau =\frac{\rho_{NR}C_{NR}l_{c}}{h},$$

where $\rho_{NR}$ is the density of NR, $C_{NR}$ is the specific heat capacity of NR and $l_{c}=V/A$ is a characteristic length that measures the sample volume *V*-to-area *A* ratio.

Note that since volume changes inNR under stretching are very small $l_{c}=\lambda^{-1/2}\cdot V_{0}/A_{0}$, where $V_{0}/A_{0}=\frac{bc}{2(b+c)}$ is the volume-to-area ratio of the unstretched sample. Hence, stretching favours the effect of convection since it decreases $l_{c}$. This is an advantage of using convection as a heat exchange mechanism in the stretched sample after crystallisation.

**Figure 6:**
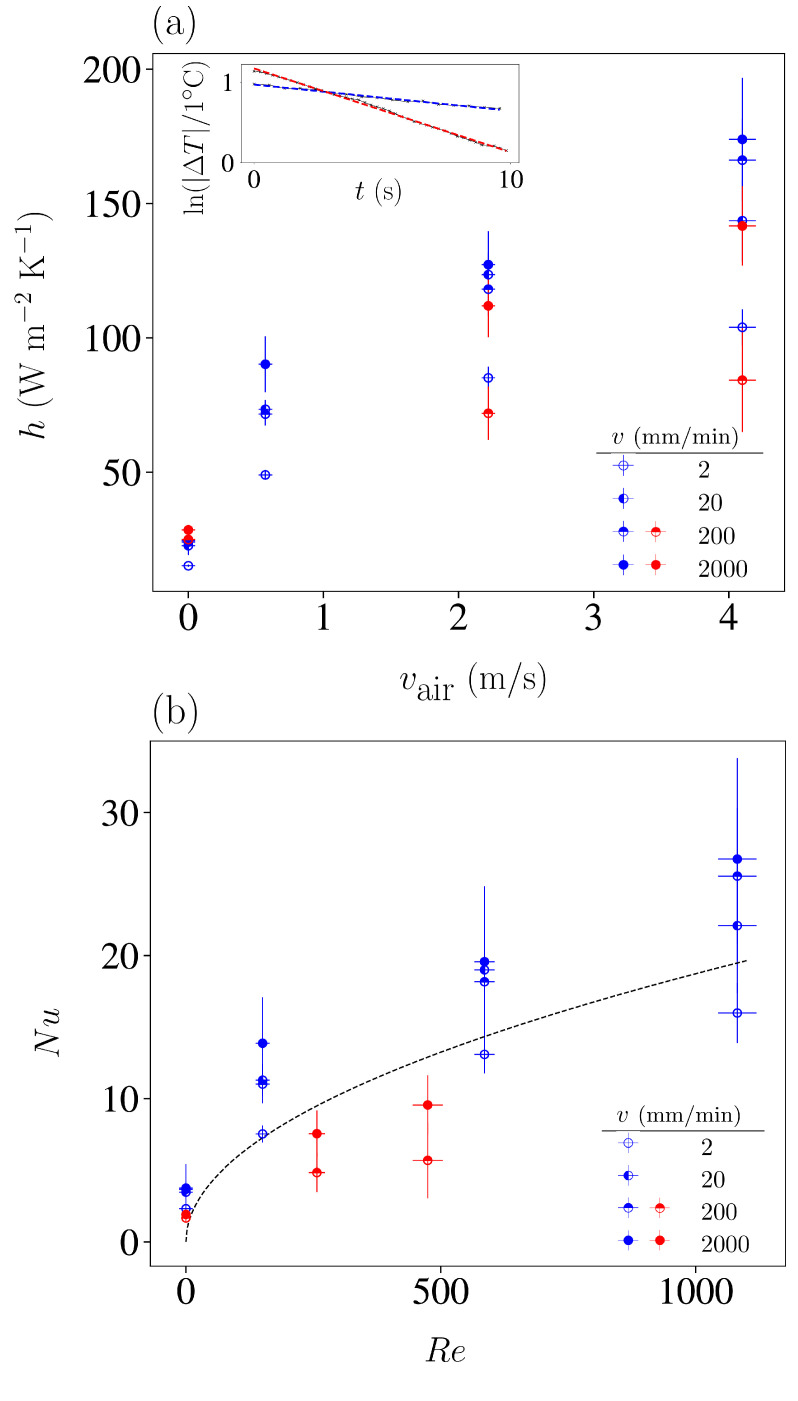
(**a**) Estimated Newton convection coefficient for NR stretched at various loading velocities and subjected to different air velocities, acting as a sink (blue) and as a source of heat (red). Vertical error bars were estimated by considering the propagation of the uncertainties in the room-temperature determination that propagate when fitting Equation (2). The inset shows examples of the fitted curves for the temperature change $\Delta T\equiv T\left(t\right)-T_{0}$ corresponding to long thermal relaxations after a loading stage with v=200 mm/min and no air convection. (**b**) The same data are represented as the Nusselt number Nu as a function of the Reynolds number Re, as explained in the text. The dashed line shows the standard relation between these adimensional numbers proposed for forced convection.

The main panel of Figure 6a compiles the obtained values of *h* as a function of air velocity. Values indicated with red symbols correspond to long pauses with constant air velocity after stretching at velocity *v*. Blue symbols correspond to experiments in which the sample was stretched at selected stretching velocities and subsequently unstretched to different undercooled states. To calculate the value of *h* based on the fitted values of τ, we used $l_{c}=\lambda^{-1}V_{0}/A_{0}=1.75\times 10^{-4}\;m$ for the stretched sample and $l_{c}=V_{0}/A_{0}=4\times 10^{-4}\;m$ for the unstretched samples.

Examples of the experimental fits of the exponential decay in Equation (2) are shown in the inset of Figure 6. They correspond to the thermal equilibration during long pauses without air convection ($v_{air}=0$) in two cases: the red curve corresponds to an experiment in which the sample heats up after loading at v=2000 mm/min, and the blue curve to an experiment in which the sample first heats up after loading at v=2000 mm/min and is subsequently unstretched at an undercooled state. Although the uncertainties in the fit of these exponential decays are small, we also considered the uncertainties in the determination of $T_{0}$ in order to estimate the error bars in *h*. Note that the different cases correspond to fits in which the initial states have different temperatures $T_{i}$ as a consequence of the internal elastocaloric heating or cooling.

An increase in air velocity consistently results in a significant rise in the coefficient *h*. For $v_{air}>$ 4 m/s, the gain in *h* is typically larger than 100% in comparison to that in the scenarios without forced air convection, and for $v_{air}=4\;m/s$, they top values of 600%.

Figure 6b displays the same data using the standard definitions of the adimensional Nusselt number and Reynolds number:

(4) $$Nu=\frac{hw}{\kappa_{air}},\;Re=\frac{v_{air}w}{\mu_{air}},$$

where $\kappa_{air}=2.7\times 10^{-2}$ ${\mathrm{W m}}^{-1}$ $K^{-1}$ is the air thermal conductivity, $\mu_{air}=1.516\times 10^{-5}$ $m^{2}$/s is the air kinematic viscosity, and *w* is a characteristic length of the forced convection problem that corresponds to the width of the sample along the air direction. For the unstretched sample (blue symbols), it corresponds to the sample width w=b=4 mm, and for the stretched sample (red symbols), it corresponds to $w=b/\sqrt{\lambda }=1.75$ mm.

The obtained Reynolds numbers are clearly below the critical value Re≃3500, which indicates that we are not in a turbulent regime. Although our experimental situation does not strictly correspond to a stationary state but rather to relaxation towards equilibrium, we can satisfactorily compare the observed trend of Nu vs. Re with the dependence:

(5) $$Nu=0.664Re^{1/2}Pr^{1/3},$$

where Pr=0.71 is the Prandtl number for air, as proposed for forced convection problems [25].

## 4. Conclusions

We conducted a series of experiments aimed at exploring the enhancement in the efficiency and the cooling power of an elastocaloric refrigeration cycle based on natural rubber. The cycles included a stretching stage carried out at different velocities, followed by an unstretching stage, always performed at maximum velocity to reach adiabatic conditions. Forced air convection was used to dissipate heat between the refrigerant and the hot reservoir.

To enhance the maximum undercooling $\Delta T_{unstr}$, it is convenient to ensure isothermal conditions are achievable during the stretching stage by driving the sample at a very low stretching velocity. However, this strategy is detrimental to the frequency of the refrigeration cycle of an operating device. Based on the limitation that the cycle frequency is generally to be maximised, it is demonstrated that an alternative approach is to implement forced air convection while in the stretching stage. At a set stretching velocity, an optimal $v_{air}$ value provides the highest level of undercooling, ΔT=−8.3 K. Furthermore, the experiments allowed for the determination of the Newton heat transfer coefficient *h* for samples that were overheated or undercooled by an internal elastocaloric effect, for different air velocities. Depending on the initial thermal conditions, the enhancement in the heat transfer coefficient can be as large as 600%. While *h* takes values around 20 W / $m^{2}$  K for $v_{air}=0\;m/s$, one can reach $h=170\pm 40\;W/m^{2}\;K$ for air velocities of $v_{air}=4\;m/s$.

These findings open up promising perspectives for the design of future elastocaloric cooling prototypes based on natural rubber using air convective heat exchange to the hot source.

## Figures and Tables

**Table 1:** Properties of the NR sample tested in this work.

Property	Magnitude	Unit	Reference
*a*	15	mm	This work.
*b*	4	mm	This work.
*c*	1	mm	This work.
$\rho_{NR}$	950	kg/m^3^	This work.
$C_{NR}$	1880	J/kg K	[7]
$\kappa_{NR}$	0.15	W/K m	[7]

**Table 2:** Adiabatic temperature difference of the elastocaloric effect of NR reported in the literature.

Reference	λ	$\Delta T_{\mathrm{unstr}}$	$\Delta T_{\mathrm{unstr}}$ with Air Convection
[7]	6	−9 °C	-
[24]	6	−4.7 °C to −5.1 °C	-
This work.	5.2	−4 °C	−8 °C

## Data Availability

The original contributions presented in the study are included in the article, further inquiries can be directed to the corresponding author.

## Nomenclature

ΔT	difference between sample surface temperature and room temperature
$\Delta T_{unstr}$	minimum difference between sample surface temperature and room temperature after fast unstretching
Δx	sample elongation
$\kappa_{NR}$	thermal conductivity
λ	local stretching ratio in the neck center
$\lambda^{total}$	total stretching ratio
$\rho_{NR}$	density
τ	characteristic time constant for thermal relaxation
*A*	sample neck surface
*a*	sample neck length
$A_{0}$	initial sample neck surface
*b*	sample neck width
*c*	sample neck thickness
$C_{NR}$	specific heat capacity of natural rubber
*d*	fan-sample distance
*F*	force
*h*	heat transfer coefficient
$L_{0}$	initial grip-to-grip length
$l_{c}$	characteristic length measuring the neck volume-to-surface ratio
Nu	Nusselt number
Pr	Prandtl number
*Q*	heat
Re	Reynolds number
*T*	average surface temperature of the sample
*t*	time
$T_{0}$	room temperature
$T_{i}$	initial temperature
*V*	sample neck volume
*v*	stretching velocity
$V_{0}$	initial sample neck volume
$v_{air}$	average air velocity
*w*	forced convection characteristic length
